# 1,1-dichloro-2,2-bis(*p*-chlorophenyl)ethylene (*p,p'*-DDE) disrupts the estrogen-androgen balance regulating the growth of hormone-dependent breast cancer cells

**DOI:** 10.1186/bcr1862

**Published:** 2008-02-14

**Authors:** Michel Aubé, Christian Larochelle, Pierre Ayotte

**Affiliations:** 1 Unité de Recherche en Santé Publique, Centre de Recherche du Centre Hospitalier Universitaire de Québec-CHUL, 2875 boulevard Laurier, Québec, QC G1V 2M2, Canada; 2 Laboratoire des biomarqueurs, Institut national de santé publique du Québec, 945 avenue Wolfe, Québec, QC G1V 5B3, Canada

## Abstract

**Introduction:**

Estrogen and androgen signalling pathways exert opposing influences on the proliferation of mammary epithelial and hormone-dependent breast cancer cells. We previously reported that plasma concentrations of 1,1-dichloro-2,2-bis(*p*-chlorophenyl)ethylene (*p,p'*-DDE), the main metabolite of the insecticide DDT (1,1,1-trichloro-2,2-bis [*p*-chlorophenyl]ethane) and a potent androgen antagonist, were associated with tumor aggressiveness in women diagnosed with breast cancer. We sought to examine the biological plausibility of this association by testing the effect of *p,p'*-DDE on the proliferation of CAMA-1 cells, a human breast cancer cell line that expresses the estrogen receptor alpha (ERα) and the androgen receptor (AR), in the presence of physiological concentrations of estrogens and androgens in the cell culture medium.

**Methods:**

The proliferation of CAMA-1 cells was determined in 96-well plates following a 9-day treatment with *p,p'*-DDE alone (0.1 to 10 μM) or in combination with 17β-estradiol (E_2_) (100 pM) and dihydrotestosterone (DHT) (100, 500, or 1,000 pM). We also assessed *p,p'*-DDE-induced modifications in cell cycle entry and the expression of the sex-steroid-dependent genes *ESR1*, *AR*, *CCND1*, and *TFF1 *(pS2) (mRNA and/or protein).

**Results:**

We found that treatment with *p,p'*-DDE induced a dose-response increase in the proliferation of CAMA-1 cells when cultivated in the presence of physiological concentrations of estrogens and androgens, but not in the absence of sex steroids in the cell culture medium. A similar effect of *p,p'*-DDE was noted on the proliferation of MCF7-AR1 cells, an estrogen-responsive cell line that was genetically engineered to overexpress the AR. DHT added together with E_2 _to the cell culture medium decreased the recruitment of CAMA-1 cells in the S phase and the expression of *ESR1 *and *CCND1 *by comparison with cells treated with E_2 _alone. These androgen-mediated effects were blocked with similar efficacy by *p,p'*-DDE and the potent antiandrogen hydroxyflutamide.

**Conclusion:**

Our results suggest that *p,p'*-DDE could increase breast cancer progression by opposing the androgen signalling pathway that inhibits growth in hormone-responsive breast cancer cells. The potential role of environmental antiandrogens in breast carcinogenesis deserves further investigation.

## Introduction

Breast cancer is the most common cancer in women, with more than 1,000,000 new cases occurring in the year 2000 worldwide [1]. Risk factors for the disease include high plasma estrogen levels [2], high levels of expression of estrogen receptors (ERs) in mammary tissue [3,4], and high breast density as revealed by mammography screening [5]. The administration of antiestrogens constitutes the most useful treatment for hormone-dependent breast cancer [6] and was shown to be effective in preventing breast cancer in clinical trials [7].

In view of the pivotal role of estrogens in the pathogenesis of breast cancer, exposure to xenobiotics that possess estrogenic properties, referred to as xenoestrogens, has been suggested to explain the increase in the incidence of breast cancer noted over the last four decades in industrialized countries. *In vitro *studies revealed that the loss of normal cell cycle control in hormone-dependent breast cancer cells can result from treatment with xenoestrogens as indicated by increased cell proliferation and modulation of estrogen-sensitive molecular parameters [8,9]. However, the sum of evidence from several epidemiological studies that investigated the relationship between breast cancer and exposure to persistent organochlorines, some of them with known estrogenic properties, does not support a link between any of these compounds and breast cancer risk [10,11].

Environmental compounds that bind the androgen receptor (AR) constitute another class of endocrine disruptors that have received growing interest over the last decade [12,13]. Androgens control the proliferation of mammary epithelial cells in nonhuman primates [14,15] as well as that of several breast cancer cell lines [16,17]. Androgens were shown to be effective in complementing the treatment of hormone-dependent breast cancer [18]. Furthermore, androgenic compounds can induce a remission after failure of antiestrogenic therapy (reviewed in [19]). One, therefore, may anticipate that exposure to antiandrogens could increase breast cancer risk or favor its progression.

1,1-dichloro-2,2-bis(*p*-chlorophenyl)ethylene (*p,p'*-DDE), the main DDT (1,1,1-trichloro-2,2-bis [*p*-chlorophenyl]ethane) metabolite, is a highly persistent molecule that accumulates in body fat with age [20] and is a potent androgen antagonist [12]. In the course of a case-control study on organochlorine and breast cancer, we previously reported that, among cases, plasma *p,p'*-DDE concentrations were associated with the aggressiveness of breast cancer [21]. We speculated that this relationship could be explained by the antiandrogenic action of the compound on breast cancer cells that would favor their proliferation and in turn breast cancer progression. To test this hypothesis, we used CAMA-1 breast cancer cells cultivated in the presence of physiologically relevant concentrations of sex hormones as an *in vitro *model of breast cancer progression. Both ER alpha (ERα) and AR are expressed in CAMA-1 cells; estrogens stimulate their proliferation, whereas androgens oppose the estrogen-induced proliferative effect [22]. Here, we show that *p,p'*-DDE can markedly increase the proliferation of CAMA-1 cells in conditions in which estrogens and androgens are competing for the control of cell cycle gene expression.

## Materials and methods

### Reagents

17β-estradiol (E_2_) was purchased from Sigma-Aldrich (St. Louis, MO, USA) and dihydrotestosterone (DHT) from Steraloids, Inc. (Newport, RI, USA), whereas hydroxyflutamide (OHF) was kindly donated by Schering-Plough Corporation (Kenilworth, NJ, USA). These compounds were dissolved in ethanol. *p,p'*-DDE was purchased from Cerilliant Corporation (Round Rock, TX, USA) and was dissolved in dimethylsulfoxide. Final concentrations of vehicles in the cell culture medium were 0.1% (vol/vol). Aprotinin, leupeptin, phenylmethylsulfonyl fluoride, and sodium orthovanadate were purchased from Sigma-Aldrich.

### Cell proliferation assays

CAMA-1 and MCF-7 cells were purchased from the American Type Culture Collection (Manassas, VA, USA). MCF7-AR1 cells were kindly provided by Ana M Soto (Tufts University, Medford, MA, USA). CAMA-1 cells were maintained in phenol red-free Roswell Park Memorial Institute (RPMI) medium supplemented with 10% (vol/vol) fetal bovine serum (FBS) from Wisent Inc. (St.-Bruno, QC, Canada), 1.0 mM pyruvate, 2.0 mM L-glutamine, 0.1 μg/mL streptomycin, and 0.1 U/mL penicillin in a humidified atmosphere of 5% CO_2 _at 37°C. Two thousand cells per well were seeded in 200 μL phenol red-free RPMI-10% FBS in 96-well plates (6 wells per treatment) and were incubated during 24 hours at 37°C. The complete medium was then substituted for FBS-free medium for a 24-hour period. On day 1 of the experiment, the FBS-free medium was replaced by a medium containing 10% dextran-coated charcoal-treated FBS (DCC-FBS) from Wisent Inc., the hormones, and test chemicals (or vehicles). Cells were grown over a 9-day period with a medium replacement every 3 days. The medium was then removed and nucleic acids were stained using the CyQuant^® ^kit purchased from Molecular Probes Inc. (now part of Invitrogen Corporation, Carlsbad, CA, USA) as described by the manufacturer. Cell proliferation for the control treatment was arbitrarily set at 1, and results were expressed as fold induction over the control.

MCF-7 and MCF7-AR1 cells were maintained in phenol red-free Dulbecco's modified Eagle's medium (DMEM) supplemented with 10% (vol/vol) FBS, 1.0 mM pyruvate, 2.0 mM L-glutamine, 0.1 μg/mL streptomycin, 0.1 U/mL penicillin, and 1 μg/mL insulin in a humidified atmosphere of 5% CO_2 _at 37°C. One thousand cells per well were seeded in 200 μL of phenol red-free DMEM-10% FBS in 96-well plates (6 wells per treatment) and were incubated during 24 hours at 37°C. The complete medium was removed and cells were washed with phosphate-buffered saline (PBS). Then a medium containing 10% DCC-FBS, the hormones, and test chemicals (or vehicles) were added. Cells were grown over a 6-day period without medium replacement, and proliferation was assessed as described above for CAMA-1 cells.

### Cell cycle analysis

Fifty thousand cells per well were seeded in 1 mL phenol red-free RPMI-10% FBS in 24-well plates and incubated during 24 hours at 37°C. The medium was replaced by FBS-free medium during 48 hours to promote G0/G1 synchronization [23]. FBS-free medium was then replaced by a medium containing 10% DCC-FBS, hormones, and test chemicals (or vehicles) for a 24-hour incubation period at 37°C. Cells were harvested following trypsinization, fixed in 70% ethanol for 30 minutes at -30°C, and stained with propidium iodide (50 μg/mL) in PBS containing 40 U/mL RNase A for 1 hour at 37°C. The DNA content in each cell was determined by flow cytometry analysis using the Wallac 1420 Multilabel Counter from PerkinElmer Life and Analytical Sciences, Inc. (Waltham, MA, USA).

### Gene expression levels

Two million cells were seeded into 10-cm dishes in 10 mL of phenol red-free RPMI-10% FBS and were incubated during 24 hours at 37°C. The complete medium was then substituted for FBS-free medium for a 24-hour period. The FBS-free medium was subsequently replaced by a medium containing 10% DCC-FBS, the hormones, and test chemicals (or vehicles), and cells were grown over a 24-hour period. Duplicate cell cultures were used for each treatment: one dish was used for RNA and the other for total cell extracts. RNA was isolated with TRIzol^® ^from Gibco (now part of Invitrogen Corporation) as described by the manufacturer and diluted in 40 μL of diethyl pyrocarbonate-treated H_2_O. mRNAs were reverse-transcribed by Super Script II™ using Oligo(dt) primer from Invitrogen Corporation as described by the manufacturer in a final volume of 50 μL. An amount of 500 ng of total RNA was included as template for each reaction. The amount of cDNA used for polymerase chain reaction (PCR) was adjusted for each target gene. To assess *ESR1 *mRNA (forward primer: 5'-AATTCAGATAATCGACGCCAG-3'; reverse: 5'-GTGTTTCAACATTCTCCCTC-CTC-3'; annealing temperature (Tm) = 58°C; 344 base pairs [bp]) [24], a 10-μL aliquot of cDNA was used compared with 1 μL for *β-actin *(forward primer: 5'-CGTGACATTAAGGAGAAGCTGTGC-3'; reverse: 5'-CTCAGGAGGAGCAATGATCTTGAT-3'; Tm = 58°C; 375 bp) [25], while 10 and 5 μL of amplified product were loaded on an 8% polyacrylamide gel for *ESR1 *and *β-actin*, respectively. To evaluate mRNAs for *CCND1 *(forward primer: 5'-CGGAGGAGAACAAACAGATC-3'; reverse: 5'-GGGTGTGCAAGCCAGGTCCA-3'; Tm = 55°C; 350 bp) [26] and AR (forward primer: 5'-GTCAAAAGCGAAATGGGCCCC-3'; reverse: 5'-CTTCTGGGTTGTCTCCTCAGT-3'; Tm = 60°C; 420 bp) [27], we used 5-μL aliquots of cDNA for both genes and a 2-μL aliquot for *β-actin *while 10 μL of amplified products was loaded on the gel. To evaluate mRNAs for *TFF1 *(forward primer: 5'-TTTGGAGCAGAGAGGAGGCAATGG-3'; reverse: 5'-TGGTATTAGGATAGAAGCACCAGGG-3'; Tm = 58°C; 240 bp) [28], we used 2-μL aliquots of cDNA and a 2-μL aliquot for *β-actin *while 10 μL of amplified products was loaded on the gel. Taq DNA polymerase and deoxynucleotides (Roche Diagnostics, Basel, Switzerland) were used as described by the manufacturer in a 50-μL final volume. The PCR settings were adjusted to complete each reaction within the linear portion of amplification. PCR conditions were one 5-minute cycle at 95°C, 25 (*β-actin*) or 30 cycles (target mRNAs) each comprising a 30-second step at 95°C, followed by a 30-second step at primer-specific Tm and a 45-second step at 72°C, and one last cycle of 7 minutes at 72°C. Negative controls were included for each reaction. PCR products were stained with ethidium bromide and captured with a 16-bit camera. Densitometry was determined by Quantity One 1-D Software Analysis from Bio-Rad Laboratories, Inc. (Hercules, CA, USA) and normalized with *β-actin*.

### Immunoblotting

Floating cells were recovered with the medium and pooled with the adherent cells that were harvested by scraping in 2 mL of ice-cold PBS, centrifuged, and resuspended in 600 μL of lysis buffer containing 50 mM Hepes, pH 7.5; 1 mM EGTA (ethylene glycol tetraacetic acid), pH 8; 150 mM NaCl; 1.5 mM MgCl_2_; 10 mM sodium pyrophosphate; 200 μM sodium orthovanadate; 100 mM NaF; 1% Triton X-100; 10% glycerol; and a protease inhibitor cocktail from EMD Biosciences, Inc. (San Diego, CA, USA). Insoluble material was removed by centrifugation (10 minutes at 13,000 *g*). Thirty micrograms of the cellular extract was resolved on PROTEAN^® ^II (Bio-Rad Laboratories, Inc.) 10% SDS-polyacrylamide gels. The proteins were electroblotted onto 0.45-μM polyvinyl difluoride membranes purchased from Millipore Corporation (Billerica, MA, USA). Membranes were blocked at room temperature for 1 hour in PBS containing 5% (wt/vol) dried milk and incubated 2 hours at 37°C with the specific antibody diluted in PBS containing 1% (wt/vol) dried milk. Antibodies against ERα, AR, and cyclin D1 were purchased from Santa Cruz Biotechnology, Inc. (Santa Cruz, CA, USA), and anti-actin was from Cedarlane Laboratories Limited (Burlington, ON, Canada). Membranes were washed in PBS containing 0.1% (vol/vol) Tween 20 followed by a 1-hour incubation with specific immunoglobulin G horseradish peroxidase-conjugated antibodies from Jackson ImmunoResearch Laboratories, Inc. (West Grove, PA, USA) and then incubated in Immun Star HRP Substrate (Bio-Rad Laboratories, Inc.) as described by the manufacturer. Signals were analyzed as described above for reverse transcription-polymerase chain reaction and were normalized for actin within the same membrane according to the method of Liao and colleagues [29].

### Statistical analyses

Concentration-response relationships were tested using linear regression analysis. Group means were compared using an analysis of variance (ANOVA) with specific contrasts or an ANOVA followed by the Bonferroni *post hoc *test. One-tail tests were performed for cell proliferation experiments because of *a priori *hypotheses regarding treatment effects (that is, inhibition for androgens and induction for antiandrogens). All other tests were two-sided. All statistical analyses were performed using the SPSS for Windows software (version 11.5.0; SPSS Inc., Chicago, IL, USA).

## Results

### Level of expression of ERα and AR in cell lines

**Figure 1:**
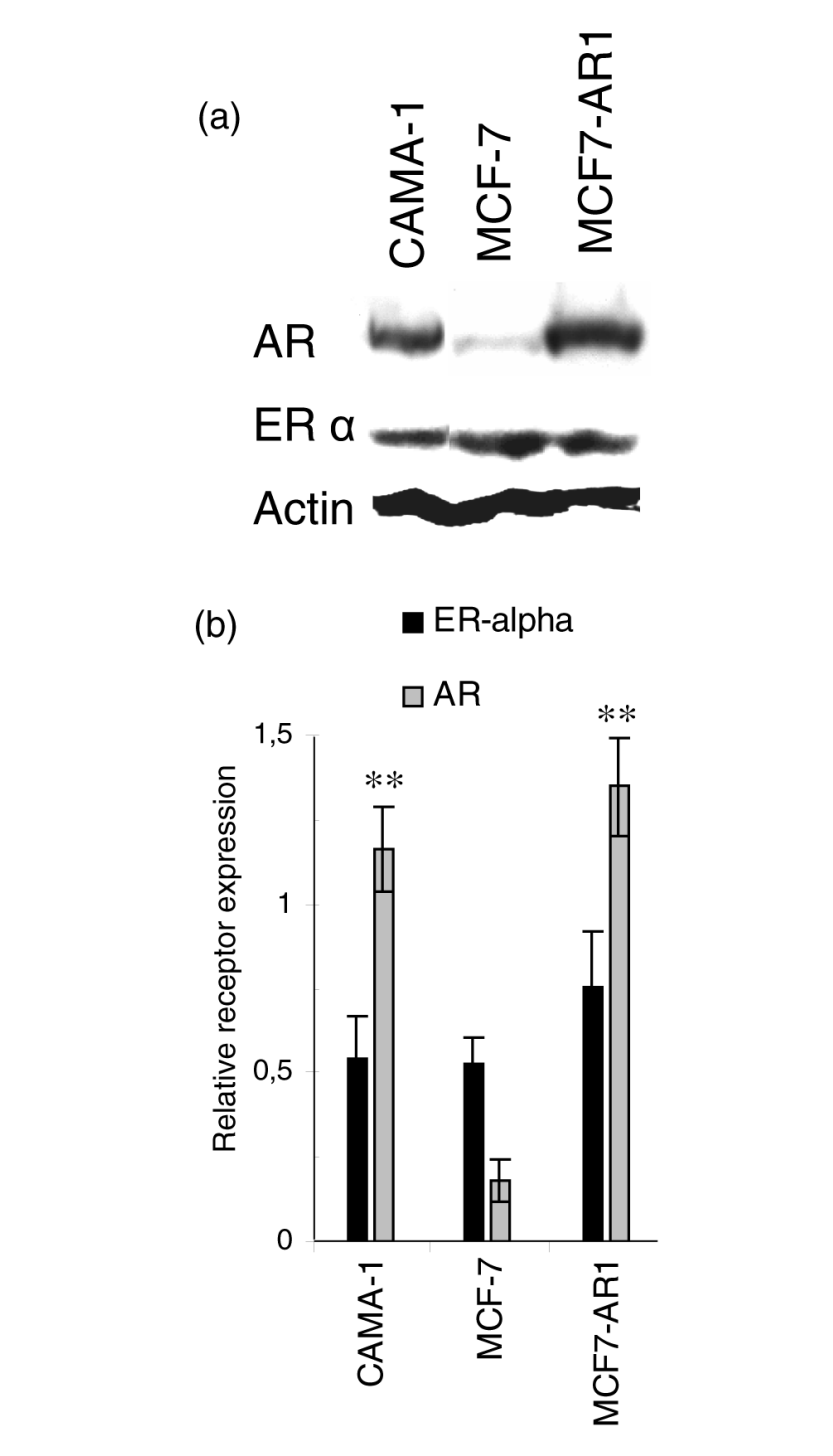
Expression of the estrogen receptor alpha (ERα) and the androgen receptor (AR) in CAMA-1, MCF-7, and MCF7-AR1 breast cancer cell lines at the protein level. Cell extracts were prepared during exponential proliferation in maintenance medium containing 10% fetal bovine serum. Immunoblots were performed as described in Materials and methods. **(a) **A representative immunoblot. **(b) **Relative expression of sex steroid receptors quantified to actin content. Each bar represents the mean ± standard error of the mean of four independent experiments. Double asterisk indicates *P *< 0.01 versus wild-type MCF-7 cells as determined by an analysis of variance followed by a Bonferroni *post hoc *test.

Figure 1 displays the relative levels of ERα and AR in CAMA-1, MCF7-AR1, and wild-type MCF-7 cells. CAMA-1 and MCF7-AR1 cells expressed, respectively, 6.5-fold (*P *< 0.01) and 7.5-fold (*P *< 0.01) more AR proteins compared with wild-type MCF-7 cells, whereas ERα expression levels were not different among the cell lines. Because of their high levels of expression of ERα and AR, CAMA-1 and MCF7-AR1 cells were used to investigate the capacity of *p,p'*-DDE to disrupt the estrogen-androgen balance and increase cell proliferation.

### Cell proliferation

**Figure 2:**
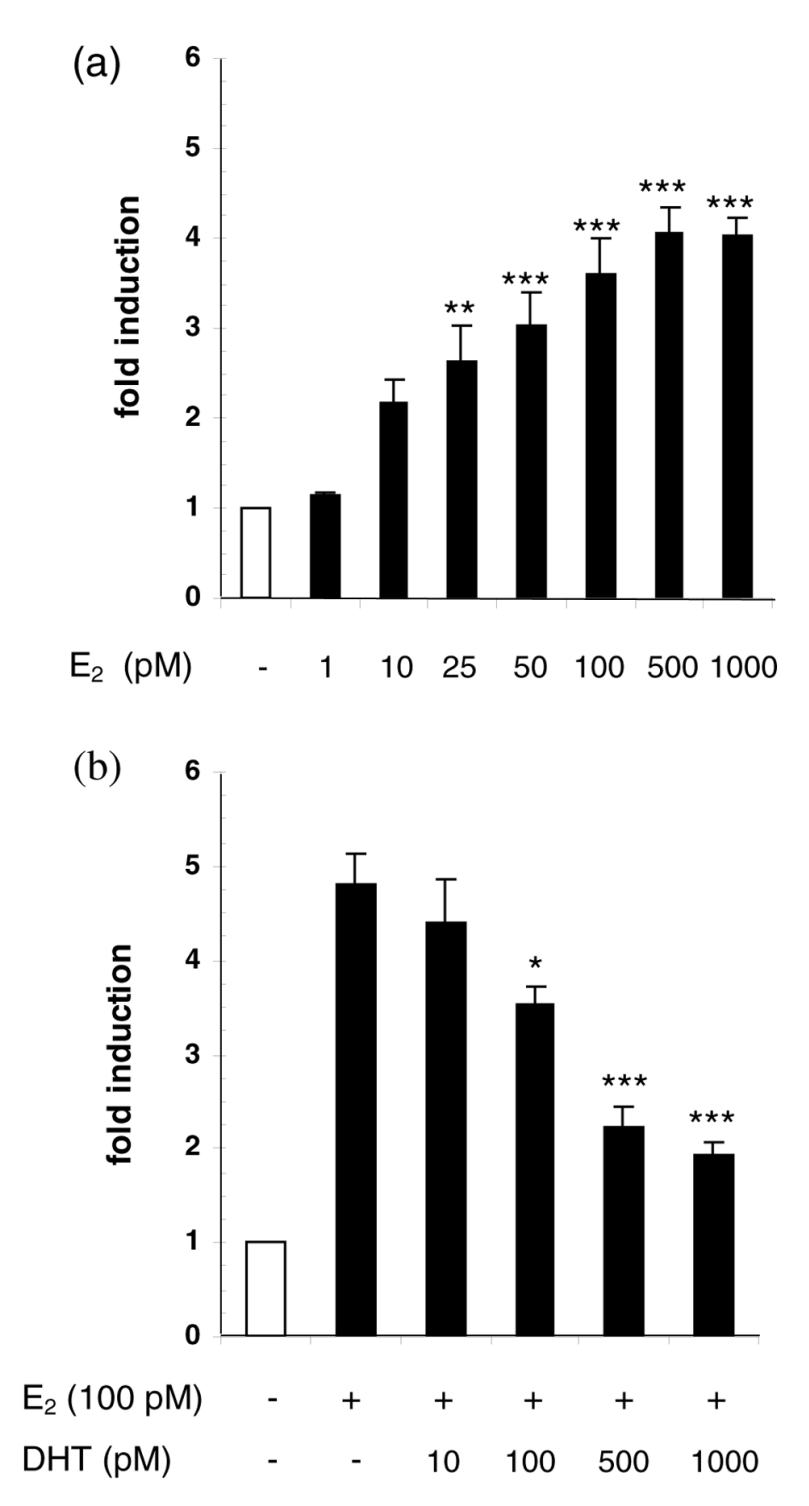
Effects of 17β-estradiol (E_2_) and dihydrotestosterone (DHT) on the proliferation of CAMA-1 cells. E_2 _induces cell proliferation in a concentration-dependent manner **(a) **while increasing concentrations of DHT inhibit the proliferative response triggered by 100 pM E_2 _**(b)**. Cell proliferation was assessed after 9 days of treatment. Each bar represents the mean ± standard error of the mean of three **(a) **and four **(b) **independent experiments. Single asterisk indicates *P *< 0.05, double asterisk *P *< 0.01, and triple asterisk *P *< 0.001 versus E_2 _treatment by an analysis of variance followed by a one-tail Bonferroni *post hoc *test.

Before testing the effect of the environmental antiandrogen *p,p'*-DDE on cell growth, we first assessed the proliferative response of CAMA-1 cells in the presence of estrogens and androgens in the cell culture medium over a 9-day period. Figure 2a shows the concentration-response relationship for E_2_-induced proliferation of CAMA-1 cells. We used a 100-pM concentration of E_2_, which generates near maximal proliferation, in combination with increasing concentrations of DHT and observed an inverse dose-response relationship between androgen concentrations (log-transformed) and cell proliferation (regression coefficient [β] = -0.887; *P *< 0.001) (Figure 2b). Combined treatment with 100, 500, or 1,000 pM DHT reduced the E_2_-induced proliferative response by 27% (*P *< 0.05), 54% (*P *< 0.001), and 60% (*P *< 0.001), respectively.

**Figure 3:**
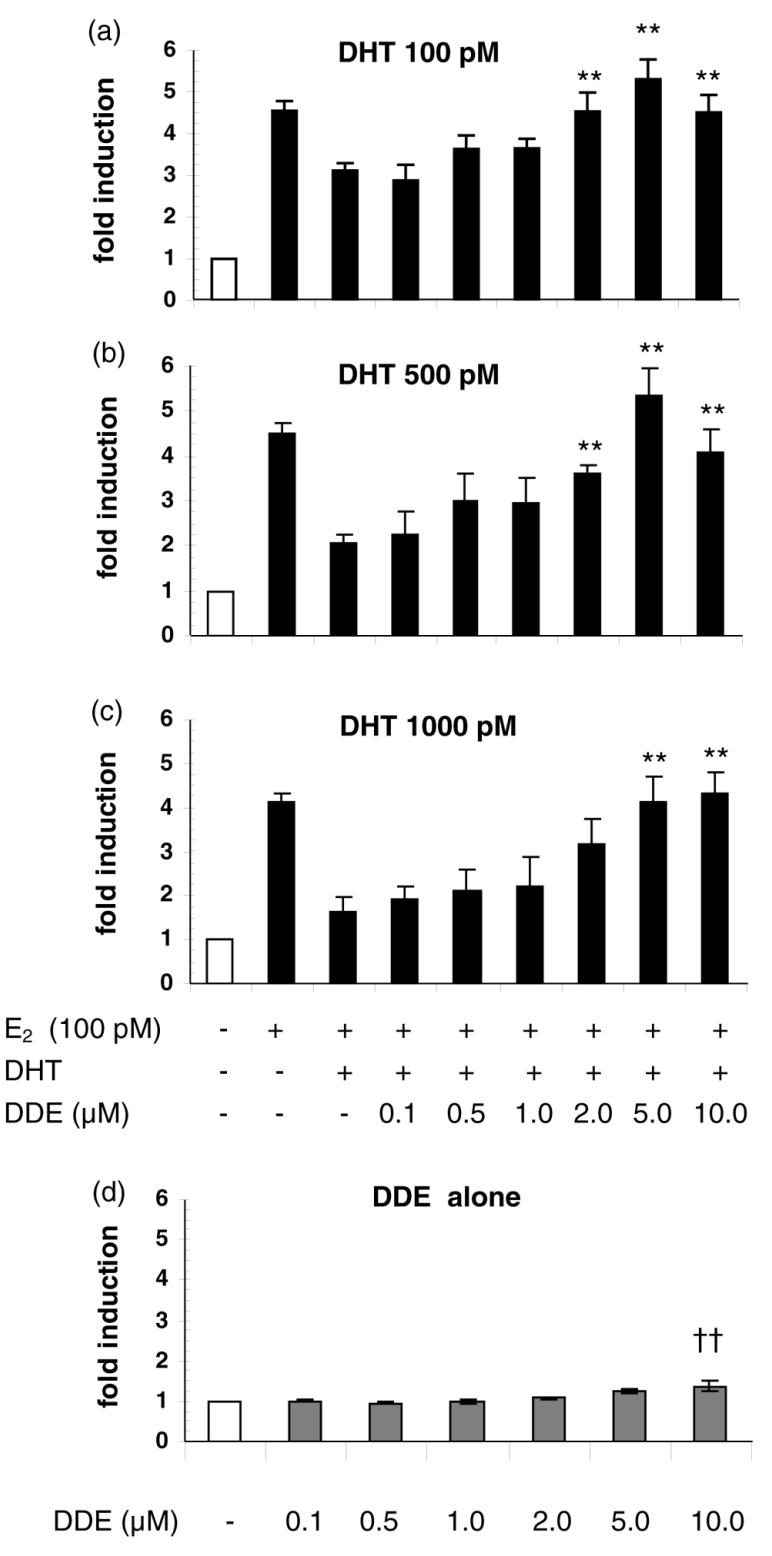
*p,p'*-DDE increases the proliferation of CAMA-1 cells in the presence of 17β-estradiol (E_2_) and dihydrotestosterone (DHT). E_2 _was added at a concentration of 100 pM, and DHT was added at concentrations of 100 pM **(a)**, 500 pM **(b)**, or 1,000 pM **(c)**. *p,p'*-DDE alone has little impact on CAMA-1 proliferation **(d)**. Cell proliferation was assessed after 9 days of treatment. Each bar represents the mean ± standard error of the mean of four independent experiments. Double asterisk indicates *P *< 0.01 versus E_2_+DHT treatment and †† indicates *P *< 0.01 versus 0.1 μM *p,p'*-DDE as determined by an analysis of variance followed by a one-tail Bonferroni *post hoc *test. *p,p'*-DDE, 1,1-dichloro-2,2-bis(*p*-chlorophenyl)ethylene.

To investigate the potential of *p,p'*-DDE to increase the proliferation of CAMA-1 cells cultivated in the presence of endogenous estrogens and androgens, increasing concentrations of *p,p'*-DDE were added to the cell culture medium together with E_2 _and DHT. *p,p'*-DDE induced concentration-related increases in CAMA-1 cell proliferation in the presence of 100 pM E_2 _and DHT added at a concentration of 100 pM (β = 0.674, *P *< 0.001; Figure 3a), 500 pM (β = 0.629, *P *< 0.001; Figure 3b), or 1,000 pM (β = 0.663, *P *< 0.001; Figure 3c). Concentrations of *p,p'*-DDE as low as 2 μM caused a statistically significant increase in cell proliferation compared with the E_2_+DHT treatment (*P *< 0.01; Figure 3a,b); the 5-μM concentration completely abolished the inhibitory effect of DHT on cell proliferation. In the absence of sex steroid hormones, *p,p'*-DDE added to the cell culture medium induced only a slight proliferative response (1.3-fold induction at 10 μM, *P *< 0.01; Figure 3d).

**Figure 4:**
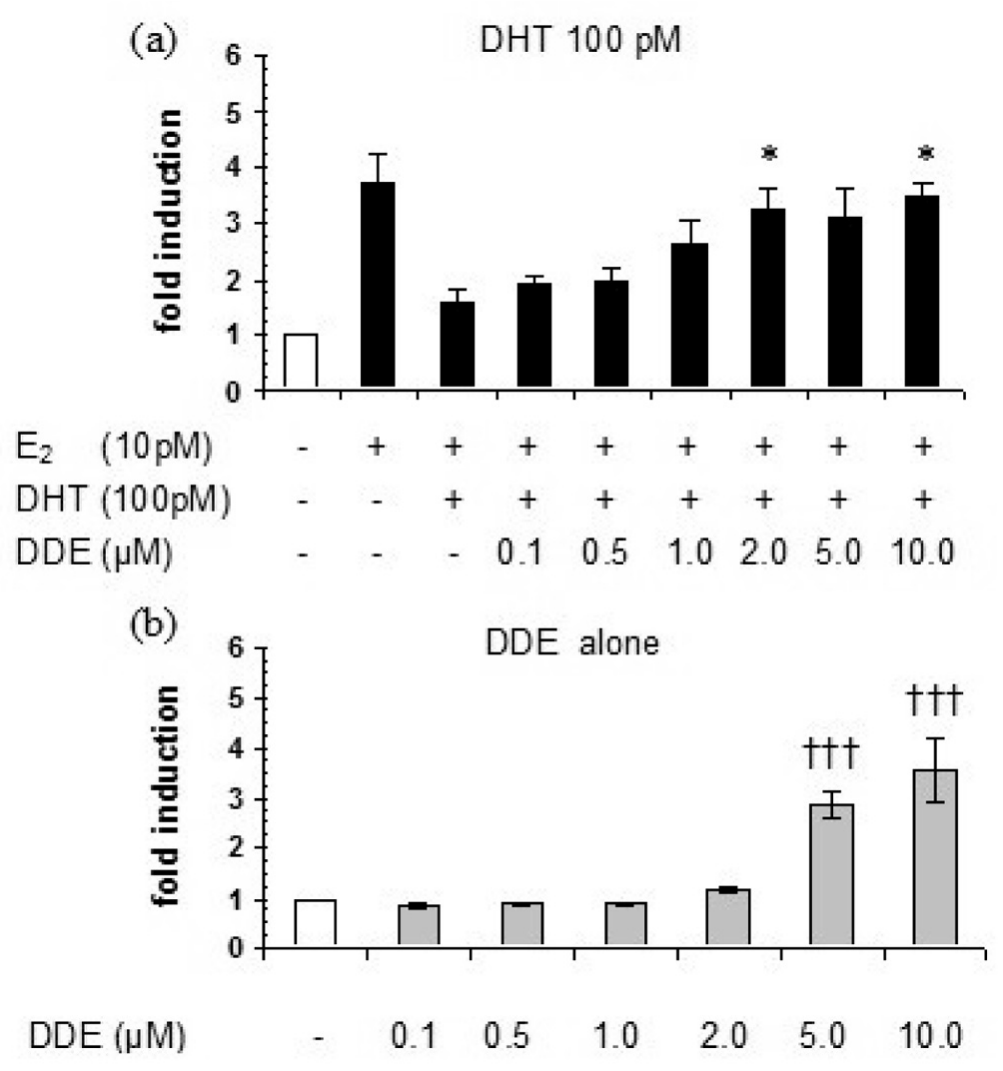
*p,p'*-DDE increases the proliferation of MCF7-AR1 cells in the presence of 17β-estradiol (E_2_) and dihydrotestosterone (DHT) **(a) **or when added alone to the cell culture medium **(b)**. Cell proliferation was assessed after 6 days of treatment. Each bar represents the mean ± standard error of the mean of four independent experiments. A single asterisk indicates *P *< 0.05 versus E_2_+DHT treatment and ††† indicates *P *< 0.001 versus 0.1 μM *p,p'*-DDE as determined by an analysis of variance followed by a one-tail Bonferroni *post hoc *test. *p,p'*-DDE, 1,1-dichloro-2,2-bis(*p*-chlorophenyl)ethylene.

The capacity of *p,p'*-DDE to increase breast cancer cell proliferation in the presence of sex steroids was also tested in MCF7-AR1 cells. Szelei and colleagues [30], who genetically engineered these cells that overexpress the AR, previously reported that DHT added together to E_2 _decreased the proliferation of MCF7-AR1 cells compared with treatment with E_2 _alone. We observed that *p,p'*-DDE induced a concentration-related increase in MCF7-AR1 proliferation in the presence of 10 pM E_2 _and 100 pM DHT (β = 0.513, *P *= 0.01; Figure 4a). A 2-μM concentration of *p,p'*-DDE caused a 2-fold increase in cell proliferation compared with the E_2_+DHT treatment (*P *< 0.05). In the absence of sex steroid hormones, *p,p'*-DDE added to the cell culture medium also induced a significant proliferative response (2.9- and 3.6-fold induction at 5 and 10 μM, respectively, *P *< 0.001; Figure 4b).

### Recruitment of CAMA-1 cells in S phase

**Figure 5:**
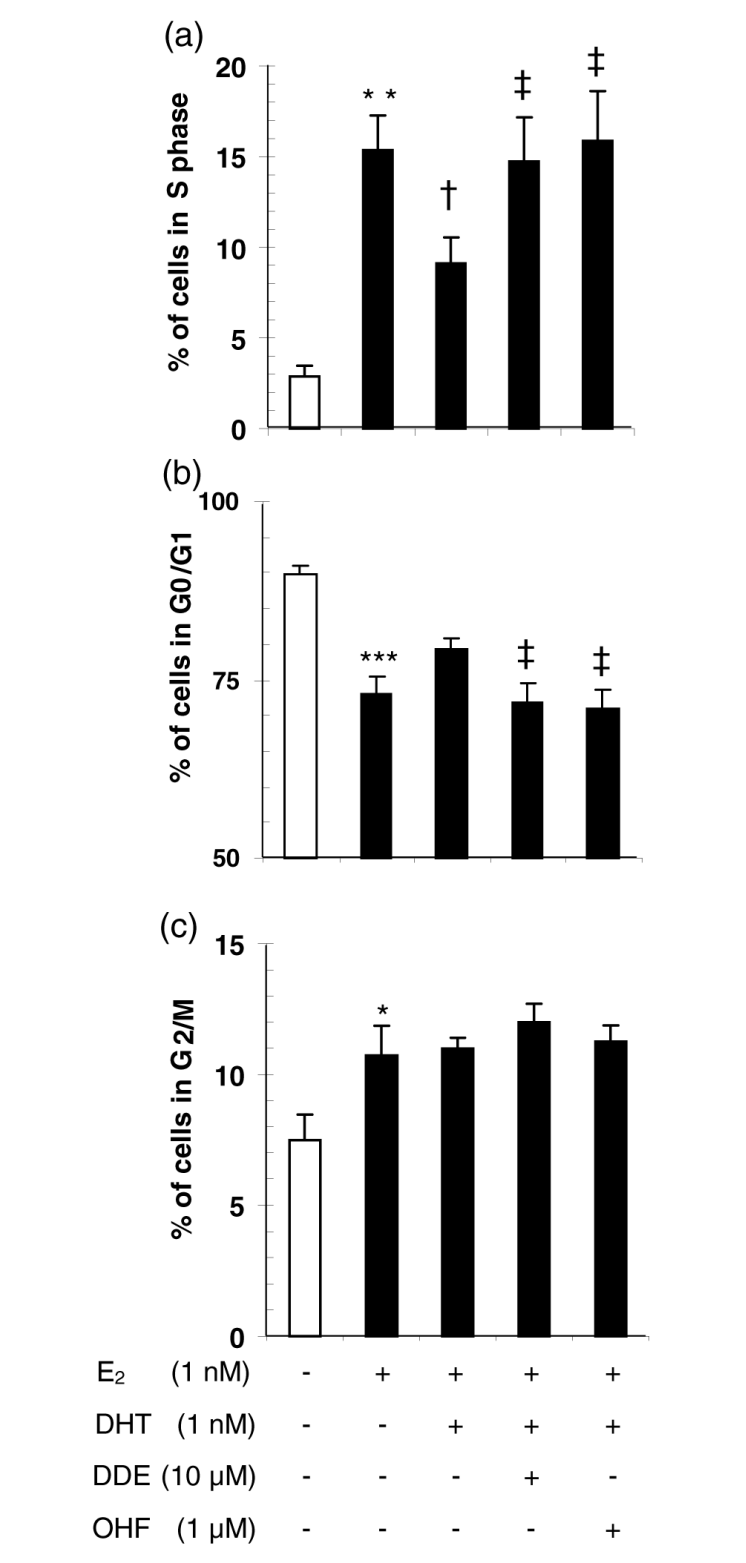
*p,p'*-DDE increases G0/G1-S transition of CAMA-1 cells in the presence of 17β-estradiol (E_2_) and dihydrotestosterone (DHT). Treatment-related changes are shown for the percentage of cells in S **(a)**, G0/G1 **(b)**, and G_2_/M **(c) **phases. Synchronization of the cells – 90% of cells in G0/G1 and less than 3% in S phase – was induced by a 48-hour serum deprivation period. Cells were subsequently treated during 24 hours with hormones and antiandrogens (or their vehicles). Each bar represents the mean ± standard error of the mean of four independent experiments in duplicate. Single asterisk indicates *P *< 0.05, double asterisk *P *< 0.01, and triple asterisk *P *< 0.001 versus control, † indicates *P *< 0.05 versus E_2 _treatment, and ‡ indicates *P *< 0.05 versus E_2_+DHT treatment as determined by an analysis of variance with specific contrasts. OHF, hydroxyflutamide; *p,p'*-DDE, 1,1-dichloro-2,2-bis(*p*-chlorophenyl)ethylene.

To better characterize the proliferative response induced by *p,p'*-DDE on CAMA-1 cells, we measured cell transition from the G0/G1 to the S phase after a 24-hour treatment with *p,p'*-DDE in the presence of sex steroid hormones and compared the results to those obtained with the potent antiandrogen OHF. Adding 1 nM DHT in combination with 1 nM E_2 _reduced by more than 50% (*P *< 0.05) the percentage of cells in the S phase observed in the presence of 1 nM E_2 _alone (Figure 5a). The addition of either 10 μM *p,p'*-DDE or 1 μM OHF to the cell culture medium completely abolished the androgen-mediated decrease in the percentage of CAMA-1 cells entering the cell cycle (*P *< 0.05 versus E_2_+DHT treatment). Treatment-induced changes in the percentage of cells in G0/G1 were of similar magnitude to those observed for the S phase but in the opposite direction (Figure 5b). The proportion of cells in the G2/M phase was slightly increased by the 1-nM E_2 _treatment (*P *< 0.05), but adding DHT and antiandrogens in combination with E_2 _did not modify the E_2_-induced response (Figure 5c).

### Modification of sex-steroid-dependent gene expression by *p,p'*-DDE

**Figure 6:**
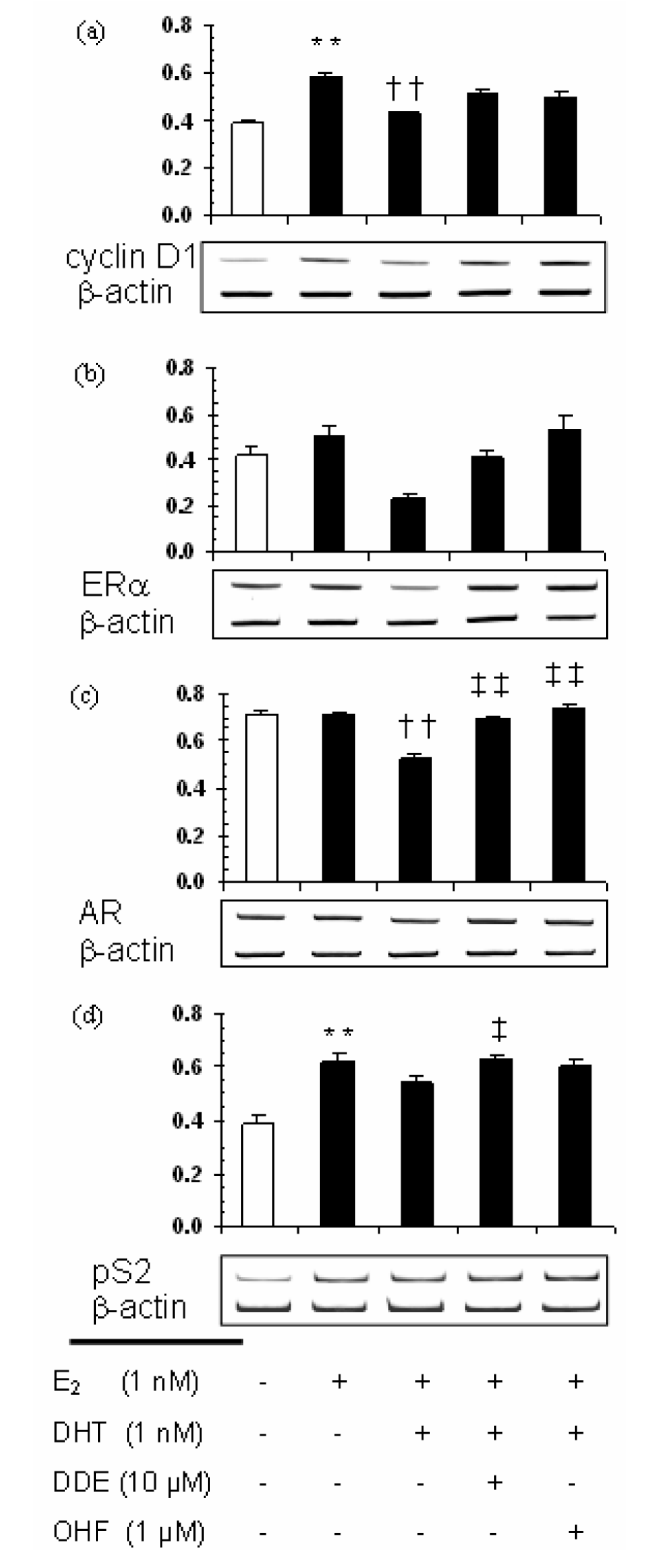
*p,p'*-DDE modulates the expression of sex-steroid-dependent genes in CAMA-1 cells at the mRNA level. mRNA levels were determined by a semiquantitative polymerase chain reaction after a 24-hour treatment with hormones and antiandrogens (or vehicles) as described in Materials and methods. mRNAs for *CCND1 ***(a)**, *ESR1 ***(b)**, *AR ***(c)**, and *TFF1 ***(d) **were quantified relative to *β-actin *mRNA. A representative gel electrophoresis is shown below each panel. Each bar represents the mean ± standard error of the mean of six independent experiments. Double asterisk indicates *P *< 0.01 versus control, †† indicates *P *< 0.01 versus E_2 _treatment, and ‡ and ‡‡ indicate, respectively, *P *< 0.05 and *P *< 0.01 versus E_2_+DHT treatment as determined by an analysis of variance with specific contrasts. AR, androgen receptor; DHT, dihydrotestosterone; E_2_, 17β-estradiol; ERα, estrogen receptor alpha; OHF, hydroxyflutamide; *p,p'*-DDE, 1,1-dichloro-2,2-bis(*p*-chlorophenyl)ethylene.

To further elucidate the mechanism underlying the induction of CAMA-1 cell proliferation by *p,p'*-DDE, we studied the effect of a 24-hour treatment with *p,p'*-DDE on the expression of sex-hormone-sensitive genes at the mRNA level, in the presence of E_2 _and DHT, and compared the results with those obtained by treating the cells with OHF in combination with the endogenous hormones. The mean cyclin D1 mRNA level was increased by 50% (*P *< 0.01) in E_2_-treated cells compared with that of the control cells (Figure 6a), whereas 1 nM DHT significantly reduced this estrogenic effect (*P *< 0.01 versus E_2 _alone). Treatment with either 10 μM *p,p'*-DDE or 1 μM OHF partly abolished the inhibition of cyclin D1 mRNA expression induced by DHT, resulting in mean expression levels that are not significantly different from that induced by E_2 _alone. Although E_2 _alone did not modulate ERα mRNA expression, the E_2_+DHT treatment appeared to decrease the mean expression level compared with that induced by E_2 _alone (Figure 6b), whereas *p,p'*-DDE or OHF partly offset this downregulation. However, differences between treatments did not reach statistical significance. AR mRNA mean expression level was decreased by 27% following DHT treatment compared with E_2 _treatment (*P *< 0.01; Figure 6c), whereas treatment with either 10 μM *p,p'*-DDE or 1 μM OHF completely antagonized this inhibition (*P *< 0.01 versus E_2_+DHT). pS2 mRNA mean expression level was increased by 50% (*P *< 0.01) following E_2 _treatment compared with the control (Figure 6d). The E_2_+DHT treatment induced a slightly lower expression of pS2 mRNA compared with that caused by E_2 _alone, but the difference was not statistically significant. *p,p'*-DDE added together with E_2 _and DHT induced a greater pS2 mRNA expression than did the E_2_+DHT treatment (*P *< 0.05).

**Figure 7:**
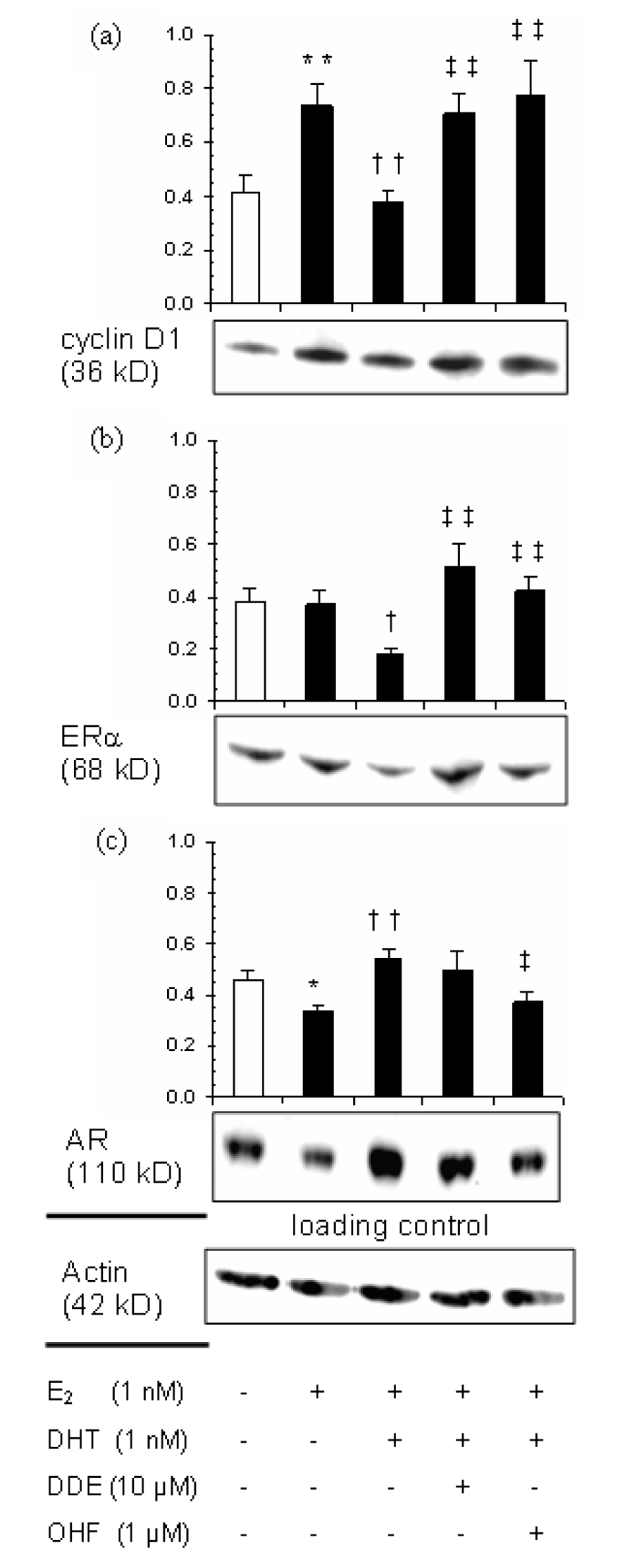
*p,p'*-DDE modulates the expression of sex-steroid-dependent genes in CAMA-1 cells at the protein level. Immunoblots were performed after 24 hours of treatment with hormones and antiandrogens (or vehicles) as described in Materials and methods. Cyclin D1 **(a)**, ERα **(b)**, and AR **(c) **protein levels were quantified relative to actin content. Each bar represents the mean ± standard error of the mean of six independent experiments. A representative immunoblot is shown below each panel. Single asterisk indicates *P *< 0.05 and double asterisk *P *< 0.01 versus control; † and †† indicate, respectively, *P *< 0.05 and *P *< 0.01 versus E_2 _treatment; and ‡ and ‡‡ indicate, respectively, *P *< 0.05 and *P *< 0.01 versus E_2_+DHT treatment as determined by an analysis of variance with specific contrasts. AR, androgen receptor; DHT, dihydrotestosterone; E_2_, 17β-estradiol; ERα, estrogen receptor alpha; OHF, hydroxyflutamide; *p,p'*-DDE, 1,1-dichloro-2,2-bis(*p*-chlorophenyl)ethylene.

We also evaluated the modulation of cyclin D1, ERα, and AR protein expression levels by *p,p'*-DDE treatment in CAMA-1 cells in the presence of endogenous sex steroids. Cyclin D1 level was increased by 80% (*P *< 0.01) in cells treated with 1 nM E_2 _compared with the vehicle-treated cells (Figure 7a), whereas adding 1 nM DHT in combination with E_2 _blocked this increase (*P *< 0.01 versus E_2 _alone). The addition of either 10 μM *p,p'*-DDE or 1 μM OHF to the cell culture medium together with E_2 _and DHT completely abolished this DHT-mediated inhibition of cyclin D1 expression (*P *< 0.01 versus E_2_+DHT). Whereas E_2 _alone was without effect, the combined E_2_+DHT treatment markedly decreased ERα protein level (more than 50%) as compared with that observed following E_2 _treatment or control (*P *< 0.05; Figure 7b). *p,p'*-DDE or OHF treatment again abolished this androgen-mediated inhibition (*P *< 0.01 versus E_2_+DHT). A different response pattern was observed for AR protein expression (Figure 7c). E_2 _treatment decreased by 28% (*P *< 0.05) the mean AR protein expression level compared with control, whereas AR protein level was significantly increased following the combined E_2_+DHT treatment compared with the value noted following E_2 _treatment alone (*P *< 0.01). The androgen-mediated increase in AR protein level was antagonized by adding OHF in the incubation medium (*P *< 0.05 versus E_2_+DHT) but not *p,p'*-DDE (Figure 7c).

## Discussion

We tested the capacity of *p,p'*-DDE to stimulate the proliferation of CAMA-1 cells, a human breast adenocarcinoma cell line that expresses both the ERα and the AR. We showed that *p,p'*-DDE strongly induces the proliferation of CAMA-1 cells in a concentration-dependent manner but only when cells are grown in the presence of physiological concentrations of endogenous sex steroid hormones. When concentrations of E_2 _and DHT are such that the androgen signalling pathway partly counteracts the influence of the estrogen signalling pathway on cell proliferation, *p,p'*-DDE blocks the AR, resulting in *CCND1 *overexpression, the recruitment of cells in the S phase, and in turn increased cell proliferation.

The capacity of the androgen DHT to inhibit the proliferation of CAMA-1 breast cancer cells was previously reported by Lapointe and Labrie [22]. Similarly to our results, these authors reported a dose-dependent inhibition of cell proliferation and maximal inhibition of E_2_-stimulated proliferation at the 1-nM DHT concentration. Other groups have reported that androgens can inhibit the proliferation of several hormone-dependent breast cancer cell lines, including MCF-7, T47D, and ZR-75-1 cells [16,17]. We tested these and other wild-type breast cancer cell lines, but in our hands only CAMA-1 cells responded strongly and reproducibly to androgens. We therefore elected to use CAMA-1 cells grown in the presence of estrogens and androgens as an *in vitro *model for investigating the role of environmental antiandrogens in breast cancer progression.

*p,p'*-DDE also induced the proliferation of MCF7-AR1 cells in the presence of E_2 _and DHT in the cell culture medium. These stably transfected cells that overexpress the AR are derived from MCF-7 cells [30], an estrogen-sensitive breast cancer cell line that has been widely used in proliferation assays for testing the estrogenic potential of chemicals (E-Screen bioassay). In contrast to results with CAMA-1 cells, *p,p'*-DDE also increased the proliferation of MCF7-AR1 cells in the absence of sex hormones (Figure 4b). This direct proliferative effect, which is likely due to the estrogenic potential of *p,p'*-DDE, was similar to that obtained by other groups with native MCF-7 cells [31-33]. Therefore, activation of the estrogenic pathway could be responsible in part for the induction of proliferation observed when MCF7-AR1 cells were cotreated with *p,p'*-DDE, E_2_, and DHT (Figure 4a). Interestingly, in the presence of E_2 _and DHT, the proliferation of MCF7-AR1 cells was induced by lower concentrations of *p,p'*-DDE than those required in the absence of sex steroids. This could be explained by the greater affinity of *p,p'*-DDE for AR than for ERα [12], resulting in the predominance of the AR signalling pathway at low concentrations. *p,p'*-DDE and several other compounds possess both antiandrogenic and estrogenic activities [34] and therefore may increase breast cancer cell proliferation through interference with both estrogenic and androgenic pathways.

Our data suggest that one of the key events in the mechanism of action through which *p,p'*-DDE increases CAMA-1 cell proliferation is the upregulation of *CCND1 *expression. Indeed, we observed concomitant increases in *CCND1 *expression and S phase entry following treatment with *p,p'*-DDE in the presence of sex steroids compared with responses induced by the E_2_+DHT treatment. This mechanism is apparently common to antiandrogens in general as similar results were observed with OHF. Cyclin D1 is a major regulator of the G1/S phase transition and a rate-limiting step in estrogen-induced mammary cell proliferation [35,36]. This oncogene has been shown to transform breast cells in transgenic mice [37] and is frequently overexpressed in primary breast cancer, especially in invasive carcinomas [38,39]. In our experiments, the cyclin D1 protein expression pattern was remarkably similar to its corresponding mRNA expression pattern, suggesting that cyclin D1 expression is mostly controlled at the mRNA level in CAMA-1 cells.

Our results also suggest that *ESR1 *expression is involved in the mechanism through which antiandrogens increase the expression of *CCND1 *in CAMA-1 cells. Effectively, we observed similar treatment-related effects for the expression of *CCND1 *and *ESR1*: DHT decreased the expression of both genes whereas treatment with either *p,p'*-DDE or OHF increased their expression in the presence of E_2 _and DHT. ERα has been shown to be an important transcription factor that acts indirectly on the *CCND1 *promoter [40-42]. That androgens can downregulate the expression of ERα was previously reported in the ZR-75-1 breast cancer cell line and in MCF7-AR1 cells [30,43].

We did not observe a reduction in ERα protein expression following treatment of CAMA-1 cells with E_2_. This result is in contrast to those reported in the literature showing that estrogens induce a downregulation of the ERα protein in hormone-dependent breast cancer cell lines as well as in transfected ER-negative cell lines [44-49]. The estrogen-induced downregulation of ERα occurs mainly through the regulated degradation of the receptor protein by the 26S proteasome [49,50]. Hence, CAMA-1 cells appear different than other breast cancer cell lines in that regard.

We found that the AR protein is downregulated by estradiol without any effect on the corresponding mRNA level. Therefore, this downregulation may occur either at the level of translation or through a decrease in AR stability. In contrast, DHT caused a significant increase in AR protein level in CAMA-1 cells. Similarly to our results, Andò and colleagues [51] observed that the activation of AR by DHT resulted in the inhibition of MCF-7 cell proliferation; this effect was accompanied by an increase in AR protein cell content.

Our results are compatible with the existence of a crosstalk between androgen and estrogen signalling pathways which controls breast cancer cell proliferation, similarly to that described by Lanzino and colleagues [52] in MCF-7 cells. These authors showed that AR activation influences ERα signalling by reducing ERα cellular content and by competition to recruit the coregulator ARA70, which although first described as a specific AR coregulator [53] also increases the transcriptional activity of ERα [52]. We speculate that binding of *p,p'*-DDE to the AR would increase the amount of ARA70 available to interact with ERα, thereby increasing the estrogenic signalling pathway and in turn cell proliferation. Additional experiments are needed to substantiate this mechanism of action in CAMA-1 cells.

Some evidence in the literature indicates that exposure to antiandrogens could increase breast cancer risk through perturbation of the androgen-estrogen crosstalk in mammary epithelial cells. Indeed, Dimitrakakis and colleagues [15] have reported an increase in mammary epithelial cell proliferation following treatment of female rhesus monkeys with flutamide (the precursor of OHF). Furthermore, a downregulation of ERα expression and a decrease in mammary epithelial cell proliferation were observed following treatment of ovariectomized rhesus monkeys with a combined estradiol/testosterone treatment compared with the group treated with estradiol alone [15]. ERα is weakly expressed in normal mammary epithelial cells and only a few cells express this gene [54], including the putative breast stem cells [55]. A rigorous control must be exerted on ERα expression in order to limit the number of 'at risk' and precancerous cells in the breast [54], which may be compromised by environmental antiandrogens.

Our results add biological plausibility to the association noted in our previous epidemiological study between plasma levels of *p,p'*-DDE and the aggressiveness of breast cancer. We observed that women with breast cancer who had higher plasma concentrations of this compound were at greater risk of having a larger tumor and axillary lymph node invasion than women with lower concentrations [21]. Although the information is extremely limited, the association between organochlorines and disease severity and progression is interesting and worthy of further investigation [11]. By blocking the androgenic pathway, *p,p'*-DDE may favor the proliferation of normal and breast cancer cells and accelerate breast cancer progression. Our results appear particularly relevant for cases with tumors expressing high levels of ERα and AR. In that context, it is worth mentioning that 70% to 90% of primary breast tumors express the AR (reviewed in [56]).

We also noted that *p,p'*-DDE increased the expression of pS2 in CAMA-1 cells (Figure 6d), an estrogen-dependent protein that increases the migration of hormone-dependent breast cancer cells [57]. The failure of OHF to increase pS2 expression over the level induced by the E_2_+DHT treatment suggests that this effect may be due to the estrogenic activity of *p,p'*-DDE. Normal breast cells secrete low levels of this chemoattractant trefoil protein [58]. This effect of *p,p'*-DDE could contribute to breast cancer aggressiveness. Additional experiments with animal models are required to further support this hypothesis.

To our knowledge, this is the first report showing that *p,p'*-DDE can significantly stimulate the proliferation of a breast cancer cell line in the presence of androgens and estrogens. Our model is unique in that compounds are tested for their capacity to stimulate cell proliferation in the presence of physiologically relevant concentrations of sex steroids. Although tests based on the proliferation of hormone-dependent breast cancer cells have been used extensively in the past, none of them can detect compounds that perturb the crosstalk between estrogenic and androgenic pathways [59]. This experimental model could be used to screen for compounds that can increase breast cancer progression because of their estrogenic potential, their antiandrogenic capacity, or a combination of both since many environmental estrogens are also AR antagonists [34].

## Conclusion

**Figure 8:**
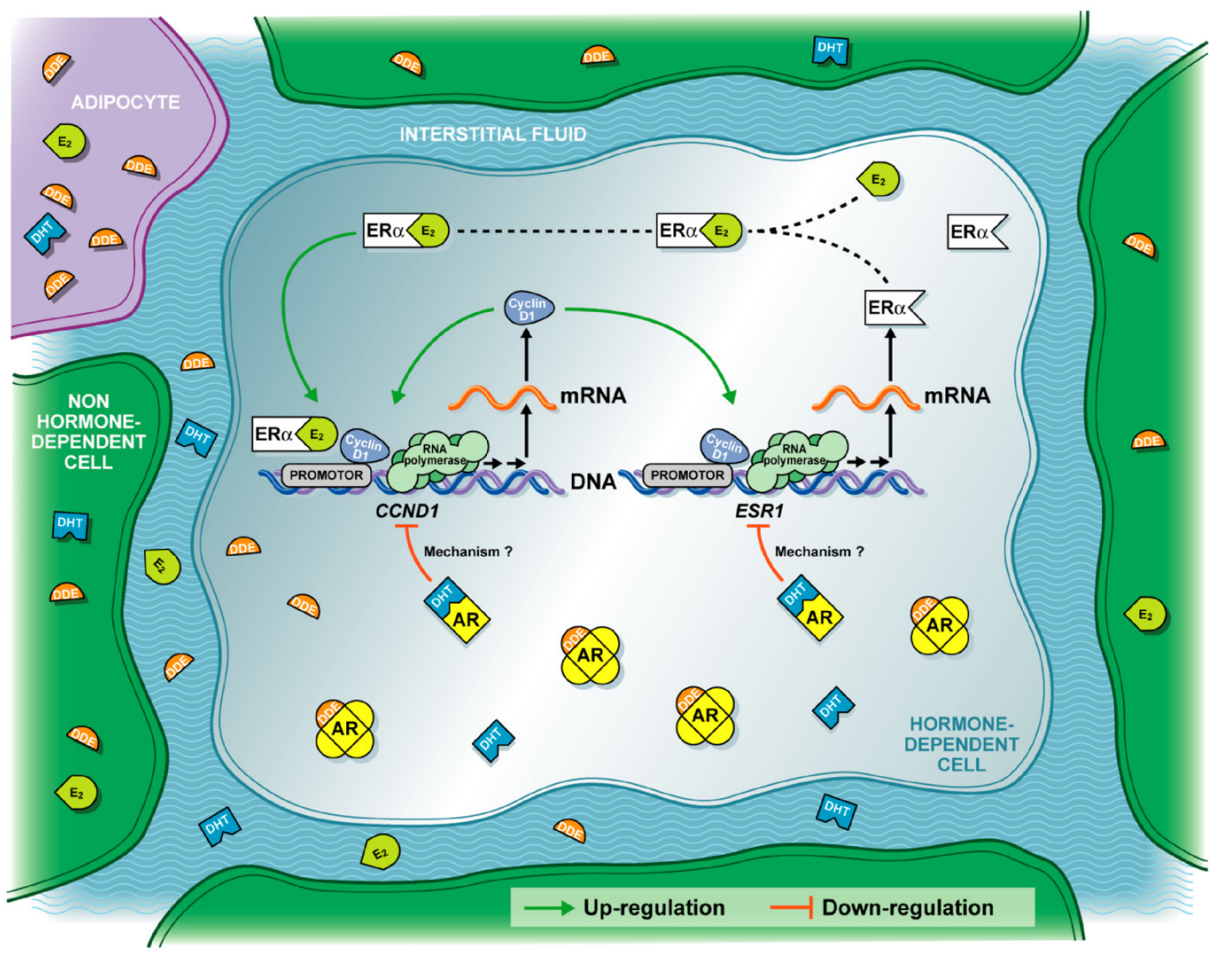
A proposed mechanism for *p,p'*-DDE-induced proliferation of hormone-dependent cells. Lipophilic *p,p'*-DDE is stored in adipocytes and can diffuse to reach hormone-dependent cells. *p,p'*-DDE confers a proliferative advantage to precancerous hormone-dependent cells by blocking the androgen receptor (AR) signalling pathway that represses cell growth. Tumor development is favored through the upregulation of the oncogene *CCND1*, a key molecular event in the deregulation by *p,p'*-DDE of the crosstalk between estrogen receptor alpha (ERα) and the AR. E_2_, 17 β-estradiol; DHT, dihydrotestosterone; *p,p'*-DDE, 1,1-dichloro-2,2-bis(*p*-chlorophenyl)ethylene.

Our study provides new evidence that environmental antiandrogens might favor breast cancer progression. Figure 8 illustrates part of the mechanism through which *p,p'*-DDE may induce the proliferation of hormone-dependent cells in the breast. Additional investigations are under way to investigate the effect on breast cancer cell proliferation of a complex mixture of environmental chemicals which comprises compounds with estrogenic and antiandrogenic activities.

## Abbreviations

ANOVA = analysis of variance; AR = androgen receptor; bp = base pairs; DCC-FBS = dextran-coated charcoal-treated fetal bovine serum; DHT = dihydrotestosterone; DMEM = Dulbecco's modified Eagle's medium; E_2 _= 17β-estradiol; ER = estrogen receptor; FBS = fetal bovine serum; OHF = hydroxyflutamide; PBS = phosphate-buffered saline; PCR = polymerase chain reaction; *p,p'*-DDE = 1,1-dichloro-2,2-bis(*p*-chlorophenyl)ethylene; RPMI = Roswell Park Memorial Institute; Tm = annealing temperature.

## Competing interests

The authors declare that they have no competing interests.

## Authors' contributions

MA contributed to the study design, conducted the experiments, and wrote the first draft of the manuscript. CL contributed to the study design. PA contributed to the study design, performed statistical analyses, supervised the experiments, and prepared the final version of the manuscript. All authors read and approved the final manuscript.
